# *MGMT* Promoter Methylation and *BRAF* V600E Mutations Are Helpful Markers to Discriminate Pleomorphic Xanthoastrocytoma from Giant Cell Glioblastoma

**DOI:** 10.1371/journal.pone.0156422

**Published:** 2016-06-02

**Authors:** Laura-Nanna Lohkamp, Maren Schinz, Claire Gehlhaar, Katrin Guse, Ulrich-Wilhelm Thomale, Peter Vajkoczy, Frank L. Heppner, Arend Koch

**Affiliations:** 1 Department of Neuropathology, Charité Universitätsmedizin Berlin, Germany; 2 Department of Neurosurgery and Pediatric Neurosurgery, Charité Universitätsmedizin Berlin, Germany; Queen Mary Hospital, HONG KONG

## Abstract

Giant Cell Glioblastoma (gcGBM) and Pleomorphic Xanthoastrocytoma (PXA) are rare astroglial tumors of the central nervous system. Although they share certain histomorphological and immunohistochemical features, they are characterized by different clinical behavior and prognosis. Nevertheless, few cases remain uncertain, as their histomorphological hallmarks and immunophenotypes do correspond to the typical pattern neither of gcGBM nor PXA. Therefore, in addition to the routinely used diagnostic histochemical and immunohistochemical markers like Gömöri, p53 and CD34, we analyzed if genetic variations like *MGMT* promoter methylation, mutations in the *IDH1/2* genes, or *BRAF* mutations, which are actually used as diagnostic, prognostic and predictive molecular markers in anaplastic glial tumors, could be helpful in the differential diagnostic of both tumor entities. We analyzed 34 gcGBM and 20 PXA for genetic variations in the above-named genes and found distinct distributions between both groups. *MGMT* promoter hypermethylation was observed in 3 out of 20 PXA compared to 14 out of 34 gcGBM (15% vs. 41.2%, p-value 0.09). *BRAF* V600E mutations were detected in 50% of the PXA but not in any of the gcGBM (50% vs. 0%, p-value < 0.001). *IDH1* R132 and *IDH* R172 mutations were not present in any of the PXA and gcGBM cases. Our data indicate, that in addition to the histological and immunohistochemical evaluation, investigation of *MGMT* promoter methylation and in particular *BRAF* V600E mutations represent reliable additional tools to sustain differentiation of gcGBM from PXA on a molecular basis. Based on these data specific BRAF kinase inhibitors could represent a promising agent in the therapy of PXA and their use should be emphasized.

## Introduction

Giant Cell Glioblastoma (gcGBM) and Pleomorphic Xanthoastrocytoma (PXA) are rare astrocytic neoplasms of the CNS, both with an equivalent incidence of <1% of all brain tumors[1,2]. gcGBM correspond to WHO grade IV tumors with a one-year-survival rate of less than 50%[2]. With 5 years recurrence-free survival rates of 72% PXA have a much better prognosis and therefore are classified as WHO grade II tumors[3]. In cases with high mitotic activity (5 or more mitoses per 10 HPF (high power fields)) and/or necrosis, the designation “PXA with anaplastic features” (PXA-A) is used[1,3]. Age distribution of gcGBM varies among adults between 45 and 75 years compared to PXA that occur predominantly in children and young adults[1,2]. The development of gcGBM is “de novo” and highly related to mutations of the *TP53* gene[2], whereas *TP53* mutations are rather uncommon in PXA[4–9].

Histologically, gcGBM and PXA share several features, which sometimes turns simple histomorphological differentiation into a difficult task[8]. In particular, the pleomorphic appearance combined with the presence of mono- or multinucleated giant astroglial tumor cells is a common histological feature, which aggravates the differentiation of both tumors. Reticulin fiber depositions are more characteristic for PXA but can also be present in gcGBM. Both tumors express, consistent with their glial lineage, the glial fibrillary acidic protein (GFAP), whereas other markers are reported to have distinct immunoreactivity, e.g. the endothelial marker CD34 is frequently expressed in tumor cells of PXA, respectively not in tumor cells of gcGBM[10]. Furthermore, nuclear p53 accumulation is typical for gcGBM, but not expected to be found in equal amounts in PXA[10]. Nevertheless, some cases demand additional diagnostic markers due to their untypical immunophenotype.

Detection of molecular alterations, including *O6-methylguanine DNA methyltransferase (MGMT)* promoter methylation, *isocitrate dehydrogenase-1 and -2 (IDH1/2)* mutations and *B-rapidly accelerated fibrosarcoma (BRAF)* V600E mutations, are state of the art in the diagnostic management of gliomas as they are highly associated with histologically defined glioma subtypes, have predictive relevance (*MGMT* status in malignant glioma in patients older than 60 years) and define molecular glioma subtypes (*IDH1/2* mutated gliomas), all representing substantial values for therapeutic and clinical outcome[11–14]. *MGMT* promoter methylation occurs in 40% of primary glioblastoma and is associated with an increased survival after radiotherapy and chemotherapy with temozolomide[15,16]. Further molecular phenomena, which have diagnostic and prognostic relevance, are mutations in the *IDH1* gene and its mitochondrial isoform *IDH2[17–19]*. *IDH1* mutations are present in the vast majority of low-grade diffuse astrocytoma[20,21], as well as in secondary glioblastoma[22,23]. Rearrangement, e.g. the copy number gain of the *BRAF* gene, located on chromosome 7q34, has been reported as a prevalent molecular alteration in pilocytic astrocytoma (WHO grade I) while being absent in most of other glial tumors[24–26]. Other *BRAF* alterations, in particular the T → A transversion at codon 600 with consecutive amino acid conversion from valine to glutamic acid have recently been identified in extra-cerebellar pilocytic astrocytoma, pleomorphic xanthoastrocytoma and ganglioglioma[27]. Moreover, detection of *BRAF* (V600E) mutations have been suggested to be helpful to distinguish PXA from diffuse astrocytic tumors WHO grade II, III and IV elsewhere[28]. Mutations of the *TP53* tumor suppressor gene, located on chromosome 17p13, are the most common mutations in different types of human cancer[5,6]^,^[29,30]. In particular, giant cell glioblastoma are characterized by high numbers of *TP53* mutations, respectively 75–90% of the cases[31], whereas the frequency of *TP53* mutations found in PXA is low. Therefore, the *TP53* mutations status might represent a reliable diagnostic marker in the discrimination of gcGBM and PXA[4,7–9].

The aim of this study was to analyze whether genetic changes of *MGMT*, *IDH1/2* and *BRAF* occur in PXA or gcGBM and if these genetic changes, respectively their distinct distribution pattern, could be used as molecular markers in the differentiation of these glial tumor entities.

## Materials and Methods

### Patients and tumor samples

Paraffin embedded tumor material from 20 PXA and 34 gcGBM were selected for histomorphological, immunohistochemical and molecular genetic analysis. Prior to DNA extraction, all tumors were reviewed by at least two neuropathologists and histopathological diagnoses were rendered according to the revised WHO 2007 classification criteria of CNS tumors using standard histological and immunohistochemical methods[2]. Clinical information was obtained retrospectively from chart review or from the online medical record SAP, according to the protocols of maintenance of patient’s confidentiality. For 3 cases clinical information were not available. All patients gave written consent at the date of surgical intervention for the use of their tissue within these studies. The study was reviewed and approved by the local ethics committee of the Charité-University (approval number EA2/101/08).

### Histological and immunohistochemical analysis

Hematoxylin-and-eosin (HE)- and reticulin staining was performed on 4 μm tissue sections from formalin-fixed, paraffin-embedded tumor specimens and evaluated for the presence of pleomorphic appearance including multinucleated giant tumor cells, giant tumor cells, perivascular lymphocytes, angiocentric tumor cell growth, vascular proliferates, necrosis and the extent of argyrophilic fiber networks. Moreover, calcification, hemosiderin deposition and mitotic activity (mitoses per 10 HPF) were taken into account. Reticulin fiber networks were evaluated as “positive” when not only confined to the vessel walls but present in more than 30% of the tumor matrix. Immunohistochemistry was carried out for the detection of GFAP (polyclonal, code Z0334, 1:2000, Dako, Glostrup, Denmark), MAP2 (monoclonal, code M4403, 1:10000, Sigma-Aldrich, St. Louis, USA), p53 (monoclonal, code M7001, 1:100, Dako, Glostrup, Denmark), CD34 (monoclonal, code M1765, 1:100, Dako, Glostrup, Denmark) and Ki-67 (monoclonal, code M7240, 1:100, Dako, Glostrup, Denmark), on 4 μm paraffin sections using a Ventana BenchMark XT® immunostainer (Ventana Medical Systems, Tucson, AZ) following the corresponding Ventana staining procedure. p53 positivity was defined as strong nuclear accumulation present in more than 30% of the tumor cells. For CD34-analysis, cytoplasmic staining of tumor cells was scored semi-quantitatively as described elsewhere[10]. CD34-positivity was rendered to tumors harboring more than 10% of CD34-expressing tumor cells. Evaluation of Ki-67 was performed in tumor areas with the highest Ki-67 labeling index by counting all tumor cells in one HPF.

### DNA extraction

Tumor tissues for DNA extraction for subsequent *MGMT* promoter methylation status, *IDH1/2* and *BRAF* mutation analysis was extracted from formalin-fixed, paraffin-embedded tissue, e.g. from 10 to 15 paraffin sections of 10 μm each after labeling on hematoxylin and eosin-stained sections. Contaminating necrotic debris, hemorrhage or normal brain tissue was excluded and only representative areas, showing an estimated tumor cell content of at least 80% have been chosen for molecular analysis. For DNA extraction from paraffin material the QIAamp DNA mini kit (Qiagen, Valencia, CA) was used. The kit was employed in accordance to the manufacturer’s instructions. Standardized quantitative and qualitative DNA assessment was performed with the NanoDrop ND-1000 Spectrophotometer and its corresponding software.

### PCR amplification of MGMT, IDH1/2 and BRAF

Information on the regions of interest, primer sequences and conditions for PCRs are listed in S1 Table. Each probe contained 12,5 μl Pyromark Mastermix, 2,5 μl Coral load (both purchased from Qiagen, Valencia, CA), 6 μl DNase-free water, 0,5 μl of the corresponding sequencing primer and 3 μl of the template due to manufacturer´s manual.

### MGMT methylation analysis

For assessment of the *MGMT* promoter methylation status a quantitative methylation-specific pyrosequencing technique was used, which interrogates 5 CpGs and therefore permits standardization with defined cut-off points. Cut-off points were defined in accordance to prospectively validated controls and accounted for a methylation percentage of 10% of at least two CpGs, or > 20% in one CpG. The DNA modification efficiency was ensured by triplet analysis of each tumor sample, including DNA quantification and constant positive and negative controls. As negative controls, white matter obtained from 5 non-tumorous biopsies from patients, aged between 40 and 50 years, were used. *MGMT* positive controls were used from tumor material, analyzed by bisulfite-sequencing. Prior to pyrosequencing of *MGMT*, the extracted DNA must be treated with bisulfite to guarantee restricted detection of methylated cytosine[32,33]. This was conducted using the EZ DNA Methylation kit (Zymo Research, Irvine, CA) according to the manufacturer’s protocol. From each patient 500 ng of genomic DNA were submitted for bisulfite conversion. Consecutive PCR amplification was performed using primer sequences of a predesigned quantitative assay (Qiagen, Valencia, CA). The product ID is indicated in S1 Table. 25 μl of the PCR product were used for pyrosequencing conducted on an automated PyroMark Q24 System (Qiagen, Valencia, CA), following the manufacturer’s instructions. Required, system-specific reagents and supplements (PyroMark Q96 Tests, ID 972032) were obtained from Qiagen (Valencia, CA).

Resulting data were analyzed and quantified with the PyroMark Q24 Software 2.0. Each tumor and control sample was analyzed in triplicates by individual PCR reactions using the same bisulfite preparation as template.

### Mutational analysis of the IDH1 (codon 132) and IDH2 (codon 172) genes

Mutational screening for *IDH1* was assessed with a mutation specific IDH1 antibody and by quantitative pyrosequencing. Staining for *IDH1* R132H was carried out on 4 μm tissue sections from formalin-fixed, paraffin-embedded tumor specimens using the *IDH1* R132H antibody (dianova GmbH, Germany). This antibody detects the heterozygous R132H point mutation of the *IDH1* gene, resulting in a substitution of arginine to histidine at position 132 of the amino acid sequence. For the immunohistochemical reactions, paraffin sections were dried at 80°C for 15 min and stained with the primary *IDH1* antibody with a dilution of 1:10 on a Ventana BenchMark XT® immunostainer (Ventana Medical Systems, Tucson, AZ) following the Ventana staining procedure. Immunoreaction was scored positive when tumor cells showed a strong cytoplasmatic and nuclear staining for IDH1 R132H. The presence of mutations affecting *IDH1* codon 132 and *IDH2* codon 172 were additionally assessed by pyrosequencing using the same reaction solutions, system and software as described above. The corresponding sequencing primers are given on request. Each tumor sample was analyzed in triplicates and each control sample in duplicates by individual PCR reactions. Non-neoplastic surgical biopsies of the white matter, obtained from 5 patients served as control.

### Mutational analysis of the BRAF gene (position V600)

*BRAF* mutations in codon 600 refer to a T > A transversion, resulting in an amino acid substitution of valine to glutamic acid. Pyrosequencing for the detection of this specific point mutation was performed as described above using a predesigned quantitative assay (BRAF Pyro Kit, ID 970470) from Qiagen (Valencia, CA).

### Statistical analyses

Pearson’s Chi-squared test with Yates’ continuity correction and Fisher’s exact tests were used to determine the association of categorical variables. Statistical significance was defined as p-value < 0.05. Analyses were conducted using R statistical software R, version 3.2.1 (The R Foundation for Statistical Computing, The R Foundation for Statistical Computing; http://www.R-project.org).

## Results

### Clinical, histological and immunohistochemical data

The tumor samples were obtained from 54 patients, including 34 gcGBM (20 males, 14 females, mean age at operation 54,9 years, range: 12–77) and 20 PXA (9 males, 11 females, mean age at operation: 46,6 years, range: 14–85) (Tables 1 and 2). Clinical data concerning the localization was available from all PXA and 31 of 34 gcGBM. gcGBM were mainly located in the frontal (11), temporal (8) and parietal (5) lobe. PXA showed a preferential localization in the temporal (10), followed by the frontal (5) lobe (Tables 1 and 2).

**Table 1:** gcGBM: Patients, histology, immunohistochemical and molecular markers.

	Age*/yr	Sex	Location	Histology	p53	Reticulin	CD34	MGMT	BRAF
1	77	F	temporal lobe	gcGBM	+	-	+	-	-
2	50	M	occipital lobe	gcGBM	+	-	-	-	-
3	74	M	occipito-temporal	gcGBM	+	+	-	-	-
4	56	M	temporo-mesial	gcGBM	-	-	+	-	-
5	55	M	insula	gcGBM	-	-	-	-	-
6	62	F	frontal lobe	gcGBM	+	-	+	-	-
7	59	F	parieto-occipital	gcGBM	-	-	-	+	-
8	73	M	post-central	gcGBM	+	+	+	-	-
9	52	F	cerebellum	gcGBM	+	-	-	-	-
10	45	F	not specified	gcGBM	+	-	-	-	-
11	47	M	temporal lobe	gcGBM	+	+	+	-	-
12	74	M	frontal lobe	gcGBM	-	-	-	-	-
13	73	M	occipital lobe	gcGBM	+	+	+	+	-
14	71	F	frontal lobe	gcGBM	+	+	+	-	-
15	66	F	frontal lobe	gcGBM	+	-	-	+	-
16	57	M	parietal lobe	gcGBM	+	-	-	+	-
17	42	M	central	gcGBM	-	-	-	+	-
18	53	F	falx cerebri	gcGBM	+	+	-	-	-
19	74	M	frontal lobe	gcGBM	-	-	-	-	-
20	48	F	frontal lobe	gcGBM	-	-	-	+	-
21	59	M	temporal lobe	gcGBM	+	-	-	+	-
22	65	M	not specified	gcGBM	+	-	-	-	-
23	61	M	frontal lobe	gcGBM	-	-	-	+	-
24	69	F	frontal lobe	gcGBM	+	+	+	-	-
25	49	F	frontal lobe	gcGBM	+	+	-	-	-
26	45	M	temporal lobe	gcGBM	+	-	-	+	-
27	12	M	hippocampus	gcGBM	+	+	-	-	-
28	20	F	occipital lobe	gcGBM	+	-	-	+	-
29	18	F	not specified	gcGBM	+	-	+	-	-
30	28	M	frontal lobe	gcGBM	-	-	-	+	-
31	66	F	parieto-occipital	gcGBM	+	-	-	-	-
32	56	M	parietal lobe	gcGBM	+	-	-	+	-
33	60	M	temporal lobe	gcGBM	+	+	-	+	-
34	50	M	frontal lobe	gcGBM	+	+	-	+	-

* age at timepoint of diagnosis

List of 34 cases diagnosed gcGBM, indicating age, gender and tumor location as well as the histological, immunohistochemical and molecular marker profile.

**Table 2:** PXA and PXA-A: Patients, histology, immunohistochemical and molecular markers.

Case	Age*/yr	Sex	Location	Histology	p53	Reticulin	CD34	MGMT	BRAF
1	61	F	temporal lobe	PXA-A	-	+	+	+	-
2	33	F	thalamus	PXA-A	-	-	-	-	-
3	15	F	temporal lobe	PXA	-	+	+	+	+
4	59	M	frontal lobe	PXA	+	+	+	-	+
5	15	F	central	PXA-A	-	+	+	-	+
6	66	M	temporal lobe	PXA-A	-	-	+	-	-
7	45	M	cerebellum	PXA-A	-	-	-	-	-
8	52	F	frontal lobe	PXA-A	-	+	+	-	-
9	52	F	temporal lobe	PXA-A	+	+	+	-	+
10	52	M	temporal lobe	PXA-A	-	+	+	-	-
11	54	M	temporal lobe	PXA-A	+	+	+	-	+
12	14	F	frontal lobe	PXA	-	+	+	-	+
13	85	M	frontal lobe	PXA	+	+	-	-	-
14	52	M	bifrontal	PXA-A	-	+	+	-	-
15	47	M	pons	PXA-A	-	+	+	-	-
16	60	F	temporal lobe	PXA	-	+	+	-	+
17	21	M	temporal lobe	PXA	-	+	+	-	+
18	51	F	temporal lobe	PXA-A	+	+	-	-	+
19	51	F	parietal lobe	PXA-A	-	+	-	-	-
20	30	F	temporal lobe	PXA-A	+	+	-	+	+

* age at timepoint of diagnosis

List of 20 cases diagnosed PXA, including 14 PXA-A. The distribution of age, gender, tumor location as well as histological, immunohistochemical and molecular marker profile are given case-wise.

Immunohistochemical analysis of all tumor samples was carried out for GFAP, MAP2, p53, CD34 and Ki-67. All gcGBM showed histomorphological criteria including vascular proliferations and/or focal necrosis. Dense reticulin fiber networks were focally observed in 11 of 34 gcGBM (32.4%) (Table 1, Table 3). GFAP and MAP2 positivity was found in all gcGBM, whereas nuclear p53 positivity could be demonstrated in 73.5% (Table 1, Table 3). CD34 expression was present in 9 out of 34 gcGBM (26.5%) (Table 1, Table 3) and Ki-67 indexes were elevated in all of the gcGBM, defining an index range of 10 to 30%.

**Table 3:** Comparative marker distribution in gcGBM and PXA.

	gcGBM	PXA	p-value*
***MGMT***	41% (n = 14/34)	15% (n = 3/20)	0,009
***BRAF* V600E**	0% (n = 0/34)	50% (n = 10/20)	< 0,001
**CD34**	27% (n = 9/34)	70% (n = 14/20)	0,005
**Reticulin**	32% (n = 11/34)	85% (n = 17/20)	< 0,001
**TP53**	74% (n = 25/34)	30% (n = 6/20)	0,005

* p-values were calculated using Pearson’s Chi-squared test with Yates continuity correction

Beside the histochemical and immunohistochemical markers reticulin, p53 and CD34, *MGMT* promoter methylation and in particular *BRAF* V600E mutation do represent helpful molecular markers to differentiate PXA from gcGBMs.

PXA showed reticulin fiber dispositions in 17 out of 20 tumors (85%) (Table 2, Table 3) and were equally marked positively for GFAP and MAP2. p53 positivity was less frequent in PXA (30% of cases), whereas CD34-expression was detected in 14 out of 20 PXA (70%) (Table 2, Table 3). PXA were also positively marked for Ki-67, PXA (WHO grade II) showing a proliferation index below 5%. 14 out of 20 PXA fulfilled the criteria for anaplasia (5 or more mitoses per 10 HPF) and were designated as PXA with anaplastic features. The proliferative activity was up to 10% of the tumor cells. Focal necrosis in PXA was also designated as a hallmark of anaplasia. There was no difference in localization preference between PXA and PXA-A observed.

### MGMT promoter methylation is much more frequent in gcGBM than in PXA

**Fig 1:**
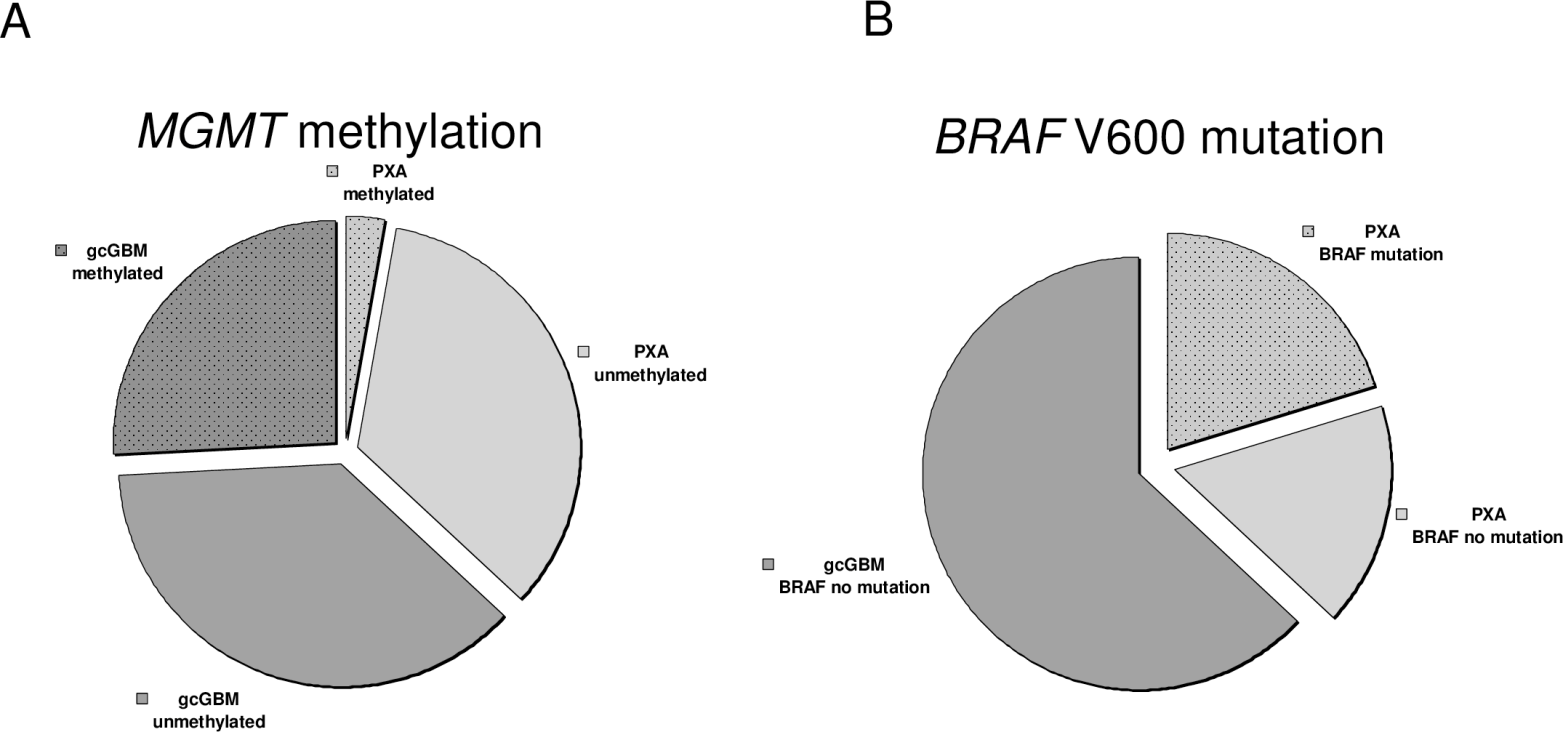
MGMT and BRAF V600 analysis in gcGBM and PXA. **A** Hypermethylation of *MGMT* was detected in 2 PXA (10%) and 14 gcGBM (41,2%). **B** 5 PXA and 6 PXA-A (55%) reveal V600E mutations in the *BRAF* gene, while being absent in gcGBM.

We examined 20 PXA and 34 gcGBM by quantitative pyrosequencing for *MGMT* promoter methylation status under standardized conditions as mentioned above. Three out of 20 PXA (15%) including two PXA which was designed as PXA with anaplastic features (PXA-A), showed a hypermethylation of the *MGMT* promoter whereas 14 out of 34 gcGBM were hypermethylated (Tables 1, 2 and 3), which corresponds to 41.2% (Fig 1A, Table 3). But statistical analysis did not confirm relevant statistical significance between both tumor entities and their methylation status (Pearson’s Chi-squared test with Yates’ continuity correction 2.88; p-value 0.09 and Fisher’s exact test p-value < 0.07, CI 95%, OR 0.26). Age and gender specific variations were not found in the groups.

### IDH1 (R132) and IDH2 (R172) point mutations are absent in gcGBM and PXA

PXA and gcGBM, were stained with a mutation specific antibody against the IDH1 residue R132H on an automated immunostainer (Ventana Medical Systems). None of the PXA or the gcGBM was marked positive. However, one PXA-A showed a focal, slight nuclear staining in the tumor cells, but not a significant positivity. Additional pyrosequencing revealed no heterozygous A to G point mutation in codon R132 of the *IDH1* gene in any of the tested PXA or gcGBM samples. *IDH2* mutations were analyzed by pyrosequencing and were also not detected in any of the groups (data not shown).

### BRAF (V600E) mutations are restricted to PXA

*BRAF* V600E mutations were equally determined by quantitative pyrosequencing, mentioning that 14 PXA were designated PXA with anaplastic features (PXA-A). A conformation change in the *BRAF* V600E gene was found in 5 PXA and 5 PXA-A in total corresponding to 50% (Tables 1, 2 and 3, Fig 1). In the gcGBM group none of the 34 samples were mutated, (Pearson’s Chi-squared test with Yates’ continuity correction 17.68; p-value < 0.001). Age and gender specific mutation rates were detected in none of the groups.

## Discussion

**Fig 2:**
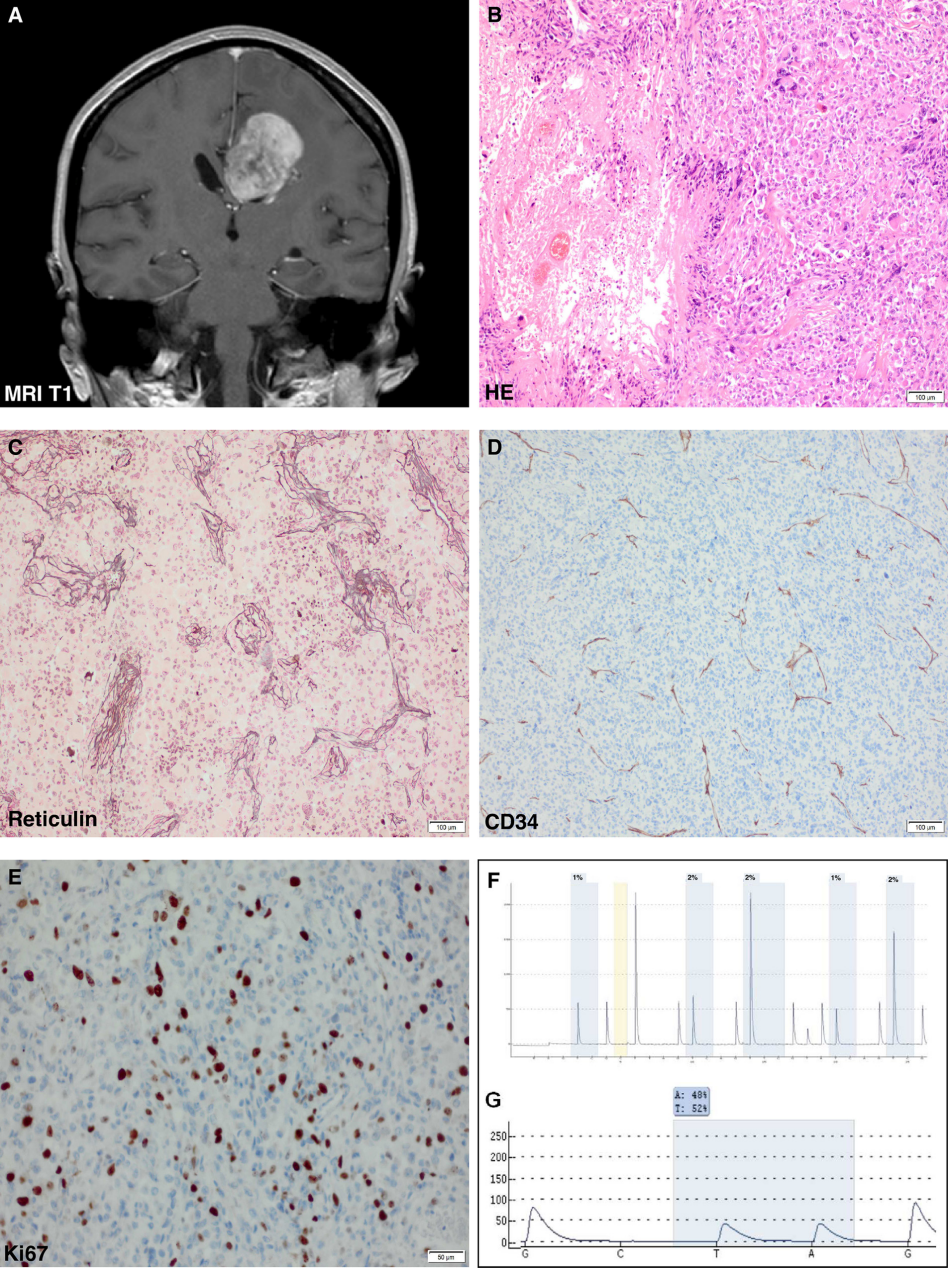
“Borderline Case” demonstrating that MGMT and BRAF are helpful additional molecular markers to differentiate PXA from gcGBM. **A** Tumor specimen of a 51-years old female patient, located in the right parietal region: T1-weighted, contrast-enhanced MRI scan shows a well-circumscribed, vascularized and in-homogeneously contrasted tumor, suspicious for focal necrosis. **B** HE staining displays a pleomorphic astroglial tumor with giant cells, elevated mitotic activity and palisading necrosis. **C** Reticulin fiber dispositions are present around the vessel walls, while **D** CD34 expression is limited to the vessels. **E** As the proliferative index additionally accounts for 20% the tumor was initially classified as giant cell glioblastoma (WHO grade IV). **F** Pyrogram indicating a significant *MGMT* hypermethylation and G *BRAF* V600E mutation. For that reason the tumor was finally classified as PXA with anaplastic features (analogues to grade III).

The differential diagnosis of PXA and gcGBM is mostly successfully answered by histochemical and immunohistochemical stainings, lightening specific hallmarks of both tumor entities. However, some cases remain uncertain due to their untypical histologic patterns and immunohistochemical marker reaction[8,34] and require additional diagnostic measures for appropriate diagnosis and consecutive personalized treatment, as experienced in the case of a 51 years old patient (compare case 19, Table 2, Fig 2). Our data indicated that in particular reticulin fiber depositions and CD34 expression, which were routinely used in PXA and gcGBM histology are robust diagnostic markers in PXA (Pearson’s Chi-squared test with Yates’ continuity correction for CD34 8.06; p-value 0,005 and for reticulin 12,88; p-value < 0,001, compare Table 3), but should be used with caution for differentiation purposes of both entities, as a dense reticulin fiber network and CD34 expression were also detected in more than 25% of gcGBM. In this context we hypothesized that genetic changes in the *MGMT*, *IDH1/2* and *BRAF* genes, which are currently considered to be most relevant for molecular diagnostics in glial tumors[11], could represent a new diagnostic tool for the integration of “boarder-line cases” based on their specific genetic fingerprints.

*MGMT* promoter hypermethylation was only found in 1 PXA and 2 PXA-A respectively. In contrast, gcGBM *MGMT* promoter hypermethylation occurred in 14 out of 34 cases, indicating prognostic and therapeutic implications and being suggestive for a prolonged survival as already described in GBM with *MGMT* promoter hypermethylation by Krex at al[35]. Other studies by Marucci & Morandi and Gömöri et al. analysed the frequency of *MGMT* promoter methylation in 11 PXA and 18 samples of primary and recurrent gliomas respectively. Marucci & Morandi detected *MGMT* promoter methylation in 2 PXA (WHO grade II), whereas none in the anaplastic variant (WHO grade III)[36]. Interestingly in our study 2 of 3 PXA with detected MGMT promoter methylation were anaplastic variants. Gömöri et al. reported present MGMT promoter methylation in both of the included gcGBM independent from tumor progression[37]. Our data emphasize that *MGMT* promoter hypermethylation is a rare epigenetic event in PXA and more typical for gcGBM. Although statistic analysis did not confirm MGMT promoter methylation as valid differential diagnostic marker (Pearson’s Chi-squared test with Yates’ continuity correction 2,88; p-value 0,009) for gcGBM and PXA, the finding of a *MGMT* hypermethylation in a PXA should be interpreted with caution and automatically lead to questioning and re-evaluation of the given diagnosis.

Using the DNA based pyrosequencing method we could confirm former studies that *BRAF* V600E mutations are a typical molecular event in PXA[28,38–40]. In contrast, we were not able to detect *BRAF* V600E mutations in any of the analyzed gcGBM demonstrating a statistically significant relevance of the *BRAF* V600E mutations in the diagnosis of PXA versus gcGBM (p-value <0.001). A high frequency of *BRAF* mutations in PXA compared to a lower frequency in gcGBM was already documented elsewhere. However, most of these studies included smaller sample sizes of one or both tumor entities or a relevantly differing research question [27,41,42]. To our knowledge this was the largest cohort of gcGBM and PXA, in which the *BRAF* V600E status was determined and therefore represents currently the most substantiated results, going in line with the previously reported findings. Another aspect of comparative study analyses is the variation of results related to distinct methods applied. For example the study of Dahiya et al. analyzed *BRAF* V600E mutations in pediatric and adult glioblastoma via immunohistochemistry using a *BRAF* V600E specific antibody. They could detect in each cohort 3 cases that were *BRAF* V600E-immunoreactive, including 2 giant cell variants in the adult group. Immunoreactivity was limited to the giant cells[41]. In our study we used a molecular approach, based on DNA sequencing, thus difficult to compare. However, this study is representative for the key issue of our study, questioning correct diagnosis of gcGBM if a *BRAF* mutation is detected. According to our results and those of previous studies[42], we emphasize that malignant astroglial tumors with histomorphological and immunohistochemical features of a giant cell glioblastoma, in which a *BRAF* V600E mutation is detected with a mutation specific antibody or by DNA sequencing should be re-examined to definitely exclude diagnosis of PXA. Another important issue is that in this study we did not analyzed the BRAF-KIAA1549 fusion as it displays a separate rearrangement with a distinct pathophysiological pathway, being predominantly found in pilocytic astrocytoma (PA). However, Antonelli et al. reported the occurrence of a KIAA1549:BRAF fusion gene in 1 anaplastic PXA, while being negative for a *BRAF* V600E mutation[43]. This finding indicates that the molecular profile of PXA is not fully elucidated yet and needs further analysis in bigger cohorts.

While *IDH1/2* mutations occur in diffuse astrocytoma, oligodendroglial tumors and secondary glioblastoma they were absent in our PXA and gcGBM samples, which differentiates PXA as well as gcGBM from diffuse astrocytoma. We could not identify *IDH1* mutations as a significant distinguishing feature of PXA and gcGBM. Moreover, putative *IDH1* mutations, which were detected with a mutation specific IDH1 antibody by immunohistochemistry, need to be interpreted carefully as only tumor samples with intense immunoreactivity were mutated within the pyrosequencing analysis.

**Fig 3:**
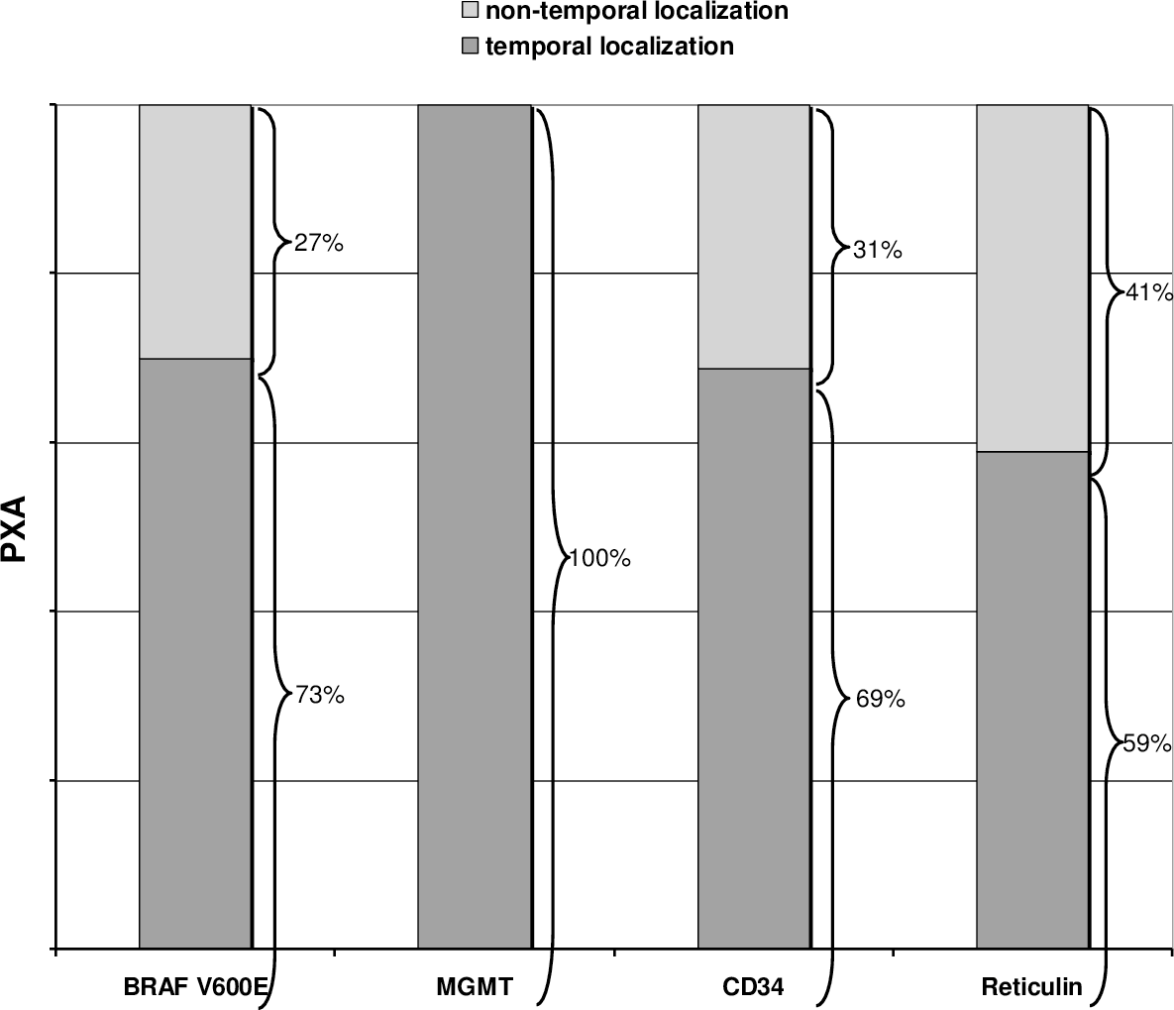
Correlation of histological and molecular markers in temporal and non-temporal located PXA. *BRAF* V600E mutations were found in 70.0% of all PXA with temporal localization (n = 7). 6 of these patients showed simultaneous reticulin and CD34 expression. Both PXA that harbored a methylation of the *MGMT* promoter were located in the temporal lobe. A slightly higher incidence of reticulin fiber disposition and CD34 expression was found in temporal tumors.

Recently, a significant coincidence of positive *BRAF* mutation status and temporal tumor localization, as well as an abundant formation of reticulin fibers in *BRAF*-mutated PXA was found by Koelsche et al.[44]. Moreover, PXA with *BRAF* mutations are highly associated with expression of the CD34-antigene[44]. In our study we could confirm a high incidence of *BRAF* mutations in temporal located PXA (7 of 10; 70%, Fig 3). Interestingly, nearly all *BRAF* mutated PXA, which were located in the temporal lobe, reveal also a dense reticulin fiber network and expressed CD34, indicating that these PXA are distinct from the PXA in non-temporal located areas, which had a heterogeneous histochemical-, immunohistochmical- and *BRAF-* profile (Fig 3). In the gcGBM group we did not detect any specific correlations between localization, histology, immunohistochemical and mutational results.

## Conclusion

In this study we could approve the diagnostic efficiency of molecular genetic analysis in the differentiation of gcGBM and PXA in the presence of atypical histological and immunohistochemical appearance. Our data reveal that *BRAF* V600E mutations do represent a common hallmark in PXA while being absent in gcGBM and therefore are a helpful molecular (diagnostic) marker to differentiate PXA from gcGBM (p-value <0,001). *MGMT* promoter hypermethylation is highly related to gcGBM and rare in PXA and therefore a well-suited, complementary diagnostic tool for the differentiation of PXA and gcGBM besides offering additional implications for targeted therapies. Being aware of the clinical and therapeutic relevance of *MGMT* promoter methylation-associated higher efficiency of temozolomide or the treatment opportunity with BRAF kinase inhibitors the investigation for *MGMT* methylation and *BRAF* V600E mutations should be implemented in the routine diagnostic of gcGBM and PXA.

## Supporting Information

S1 Table Primers and sequences. (DOCX)
