# Supplementary material for: MGMT Promoter Methylation and BRAF V600E Mutations Are Helpful Markers to Discriminate Pleomorphic Xanthoastrocytoma from Giant Cell Glioblastoma
Source: PLoS One. 2016 Jun 2;11(6):e0156422. doi: 10.1371/journal.pone.0156422 (PMC4890800; doi:10.1371/journal.pone.0156422)
Supplement: S1 Table — (DOCX) [file pone.0156422.s001.docx]

**S1 Table: Primers and sequences**

| **Gene** | **Primer sequence 5'-3'** | **PCR product** | **Tm [°C]** | **PCR cycles** |
| --- | --- | --- | --- | --- |
| ***IDH1*** | f: GCT TGT GAG TGG ATG GGT AAA | 123 bp | 58,2 | 35 |
|  | r: GTT GGA AAT TTC TGG GCC ATG |  |  |  |
|  | s: TGG GTA AAA CCT ATC ATC |  |  |  |
| ***IDH2*** | f: TTC CGG GAG CCC ATC ATC | 111 bp | 58,2 | 35 |
|  | r: CTC TCC ACC CTG GCC TAC CT |  |  |  |
|  | s: CAT CCC ACG CCT AGT |  |  |  |
| ***MGMT*** | f: Qiagen ID. 972032 | 104 bp | 58,6 | 45 |
|  | r: Qiagen |  |  |  |
|  | s: Qiagen |  |  |  |
| ***BRAF* V600E** | f: Qiagen ID. 972412 | 375 bp | 60 | 45 |
|  | r: Qiagen |  |  |  |
|  | s: Qiagen |  |  |  |
